# HOPE (SOLTI-1903) breast cancer study: real-world, patient-centric, clinical practice study to assess the impact of genomic data on next treatment decision-choice in patients with locally advanced or metastatic breast cancer

**DOI:** 10.3389/fonc.2023.1151496

**Published:** 2023-04-28

**Authors:** Rubén Olivera-Salguero, Elia Seguí, Juan Miguel Cejalvo, Mafalda Oliveira, Pablo Tolosa, Maria Vidal, Marcos Malumbres, Joaquín Gavilá, Cristina Saura, Sonia Pernas, Rafael López, Mireia Margelí, Judith Balmaña, Montserrat Muñoz, Isabel Blancas, Valentina Boni, Eva Ciruelos, Elena Galve, Antonia Perelló, Rodrigo Sánchez-Bayona, Susana de la Cruz, Miguel de la Hoya, Patricia Galván, Esther Sanfeliu, Blanca Gonzalez-Farre, Valeria Sirenko, Aura Blanch-Torras, Jordi Canes, Helena Masanas, Rosa Olmos, Margarita Forns, Aleix Prat, Ana Casas, Tomás Pascual

**Affiliations:** ^1^ SOLTI Cancer Research Group, Barcelona, Spain; ^2^ Medical Oncology Department, Hospital Clinic of Barcelona, Barcelona, Spain; ^3^ Translational Genomics and Targeted Therapies in Solid Tumors, August Pi i Sunyer Biomedical Research Institute (IDIBAPS), Barcelona, Spain; ^4^ Hospital Clínico Universitario de Valencia, INCLIVA (Instituto de Investigación Sanitaria), Universidad Valencia, Valencia, Spain; ^5^ Medical Oncology Department, Vall d’Hebron University Hospital, Barcelona, Spain; ^6^ Breast Cancer Group, Vall d’Hebron Institute of Oncology (VHIO), Barcelona, Spain; ^7^ Medical Oncology Department, Hospital Universitario 12 de Octubre, Madrid, Spain; ^8^ Faculty of Medicine and Health Sciences, University of Barcelona, Barcelona, Spain; ^9^ Breast Cancer Unit, Institute of Oncology Barcelona (IOB) – Quirónsalud, Barcelona, Spain; ^10^ Cancer Cell Cycle Group, Vall d’Hebron Institute of Oncology (VHIO), Barcelona, Spain; ^11^ Cell Division and Cancer Group, Spanish National Cancer Research Centre (CNIO), Madrid, Spain; ^12^ Institució Catalana de Recerca i Estudis Avançats (ICREA), Barcelona, Spain; ^13^ Instituto Valenciano de Oncología (IVO), Valencia, Spain; ^14^ Medical Oncology Department, Catalan Institute of Oncology (ICO)/Institut d’Investigació Biomédica de Bellvitge (IDIBELL), L’Hospitalet de Llobregat, Barcelona, Spain; ^15^ Medical Oncology Department and Translational Medical Oncology Group, Clinical University Hospital and Health Research Institute of Santiago de Compostela (IDIS)-CIBERONC, Santiago de Compostela, Spain; ^16^ Catalan Institute of Oncology (ICO), Hospital Germans Trias i Pujol/Badalona Applied Research Group in Oncology (B-ARGO Group), Oncology Department, Badalona, Spain; ^17^ Hospital Universitario San Cecilio, Instituto de Investigación Biosanitaria de Granada (ibs. Granada) and Medicine Department, Granada University, Granada, Spain; ^18^ Next Madrid, Universitary Hospital Quiron Salud Madrid, Madrid, Spain; ^19^ Centro Integral Oncológico Clara Campal HM (CIOCC), Madrid, Spain; ^20^ Hospital Universitario Basurto (OSI Bilbao-Basurto), Bilbao, Spain; ^21^ Hospital Universitari Son Espases, Palma de Mallorca, Spain; ^22^ Medical Oncology Department, Complejo Hospitalario de Navarra, Pamplona, Spain; ^23^ Molecular Oncology Laboratory, CIBERONC, Hospital Clinico San Carlos, Instituto de Investigación Sanitaria San Carlos, Madrid, Spain; ^24^ Pathology Department, Hospital Clinic of Barcelona, Barcelona, Spain; ^25^ Asociación Española de Cáncer de Mama Metastásico, Oviedo, Spain; ^26^ Fundación Actitud Frente al Cáncer, Sevilla, Spain

**Keywords:** molecular advisory board, molecular tumor board, metastatic breast cancer, genomic data, targeted therapy, patient-centric trials

## Abstract

**Background:**

Metastatic breast cancer (mBC) causes nearly all BC-related deaths. Next-generation sequencing (NGS) technologies allow for the application of personalized medicine using targeted therapies that could improve patients’ outcomes. However, NGS is not routinely used in the clinical practice and its cost induces access-inequity among patients. We hypothesized that promoting active patient participation in the management of their disease offering access to NGS testing and to the subsequent medical interpretation and recommendations provided by a multidisciplinary molecular advisory board (MAB) could contribute to progressively overcome this challenge. We designed HOPE (SOLTI-1903) breast cancer trial, a study where patients voluntarily lead their inclusion through a digital tool (DT). The main objectives of HOPE study are to empower mBC patients, gather real-world data on the use of molecular information in the management of mBC and to generate evidence to assess the clinical utility for healthcare systems.

**Trial design:**

After self-registration through the DT, the study team validates eligibility criteria and assists patients with mBC in the subsequent steps. Patients get access to the information sheet and sign the informed consent form through an advanced digital signature. Afterwards, they provide the most recent (preferably) metastatic archival tumor sample for DNA-sequencing and a blood sample obtained at the time of disease progression for ctDNA analysis. Paired results are reviewed by the MAB, considering patient’s medical history. The MAB provides a further interpretation of molecular results and potential treatment recommendations, including ongoing clinical trials and further (germline) genetic testing. Participants self-document their treatment and disease evolution for the next 2 years. Patients are encouraged to involve their physicians in the study. HOPE also includes a patient empowerment program with educational workshops and videos about mBC and precision medicine in oncology. The primary endpoint of the study was to describe the feasibility of a patient-centric precision oncology program in mBC patients when a comprehensive genomic profile is available to decide on a subsequent line of treatment.

**Clinical trial registration:**

www.soltihope.com, identifier NCT04497285.

## 1 Introduction

Breast cancer (BC) is the most prevalent solid tumor and the main cause of cancer-related deaths among females (1). Advances in high-throughput sequencing techniques, also known as next-generation sequencing (NGS) techniques, have boosted the classification of BC into distinct molecular subtypes with different clinical implications, thus leading to a paradigm shift in our understanding of the disease (2).

The discovery of new biomarkers has historically led to the development of new targeted therapies. After uncovering that an important proportion of bad prognosis BC patients overexpress the HER-2 receptor, humanized monoclonal antibodies targeting the extracellular domain of the receptor were developed, promoting a remarkable increase in life expectancy for this subgroup of patients (3, 4). More recently, after discovering the DNA-repair defects present in several familiar ovarian and breast tumors, synthetic lethal therapeutic strategies with poly (adenosine diphosphate [ADP]–ribose) polymerase (PARP) inhibitors were developed, with also a significant impact on these patients’ survival (5, 6). Similarly, the high prevalence of genetic alterations of the PI3K/mTOR pathway found in BC provided the rationale for the development of inhibitors blocking key points in this pathway. Continued efforts have been made to improve these targeted therapies ultimately achieving clinical benefit for BC patients (7, 8). The improvement in NGS techniques is catalyzing the blooming of new putative targeted agents whose efficacy must be assessed in prospective clinical trials (9). Tools such as the ESMO Scale for Clinical Actionability of molecular Targets (ESCAT) supports the interpretation of molecular alterations identified in the patient tumors (10). Results from the phase II study SAFIR02-BREAST showed that the use of multigene sequencing improved the outcomes for patients with metastatic breast cancer if they carry alterations classified as ESCAT I/II (11). However, new targeted therapies and clinical trials evaluating promising novel therapies will only be pertinent if we can properly identify patients harboring these genetic alterations in the real-world setting.

For decades, recommendations from international oncology societies have stated that the best management of any patient with cancer is the enrollment in a clinical trial. Despite this recommendation, a limited proportion of patients with mBC are enrolled in trials. One key barrier is that patients receive targeted therapies or are enrolled in clinical trials upon the analysis of only a limited number of molecular alterations. This narrow approach has the serious limitation that patients in whom no alteration can be found might be eligible to receive other targeted therapies, but the time required for any additional testing could seriously compromise their outcome. Authors agree that analyzing simultaneously multiple therapeutic targets with high-throughput sequencing techniques could overcome this limitation (12, 13). Nevertheless, there is inequitable access to biomarker testing and NGS is not routinely used in the clinics due to economic and logistic limitations.

Both the feasibility and impact of molecular screening programs to select the best targeted therapy based on high-throughput technologies are being assessed in different initiatives implicating national and international efforts (11, 14–16). In 2014 our group launched the pilot program AGATA, the first Spanish nationwide molecular screening program involving three laboratories experienced in high-throughput sequencing techniques and ten hospitals from the SOLTI network. DNA-sequencing of 56 cancer related genes was performed using formalin-fixed paraffin-embedded (FFPE) tumor samples (primary or metastatic). All clinical cases were reviewed by a Molecular Advisory Board (MAB), composed by medical oncologists, pathologists, and molecular biologists, which recommended potential novel treatments mainly in the context of a clinical trial. From September 2014 to July 2017, 305 patients were screened and 260 (85.3%) were evaluated by the MAB. AGATA detected actionable mutations in nearly half of the patients while only 11% of them subsequently received targeted therapy (17). Similar worldwide initiatives have also found this little amount of targetable mBC patients and similar final percentages of patients receiving targeted therapies based on the identified alterations (11, 16). Presumably, the 56 gene panel we used in AGATA, not covering copy number alterations or genes associated with hereditary BC, was insufficient to detect all putative targetable alterations in the study population. Moreover, participants were heavily pre-treated (median of 3 lines), which could have limited their inclusion in clinical trials with strict inclusion/exclusion criteria. Finally, the inclusion rate was relatively slow (305 patients in 34 months), and participants were referred to the study by academic medical institutions only, raising the possibility that the study was not capturing accurately the real-world scenario of all patients diagnosed with mBC in Spain.

Thus, the HOPE study was designed to overcome previous limitations of our pilot study and to draw more relevant conclusions. It follows a patient-centric (**Table 1**. Patient-centric trials *vs* classical approach) approach in which mBC patients voluntarily enroll themselves in the study and manage their participation by providing samples, clinical information, and follow-up data, independently of their treating physician and institution. The Spanish Metastatic Breast Cancer Association, an advocacy BC group was actively implicated in the study’s onset and is part of the study’s government.

**Table 1:** Patient-centric trials *vs* classical approach.

	PATIENT PARTICIPATION		CLINICAL/FOLLOW-UP DATA	SAMPLES		TRIAL OUTCOME		
	Enrollment	Inclusion/Exclusion Criteria		Logistics	Analyses	Treatment Possibilities	Patient Education	Physician Education
**Classical Trials** “Which patient fits my trial?”	Promoted by physicians (Restricted to certain regions and centers, Patient as a passive participant)	Restrictive (Selected population)	Provided by professionals (Accurate)	Center-driven (Geographical limitations)	Discrete (Targeting specific alterations)	Limited arms of treatment	Limited (Restricted to a trial)	Limited (Restricted to a disease context)
**Patient-Centric Trials** “Which trial fits my patient?”	Promoted by patients (Across regions and centers, Patient as an active partner)	Comprehensive (Real-world population)	Provided by patients (Limited, but potentially curated by a medical team)	Patient-provided (Across regions)	Comprehensive (NGS)	Recommended targeted therapies matching diverse patient contexts	Potentially high (Concept of Precision Medicine, Importance of Clinical Trials, Possibilities of NGS, Patient empowerment facing the disease)	Potentially high (Multi-centric discussion, Different disease contexts, Patient derivation between centers)

Patient-centric trials are intended to provide personalized therapies matching patient’s characteristics. They rely on cooperative networks of professionals and on the active implication of participants during enrollment, clinical and follow-up data delivery and sample obtention. Rather than using a single diagnostic test, patient-centric trials have a comprehensive design that integrate patient needs and multiplex molecular testing, allowing to “find the ideal trial/treatment for the patient”. In addition, patient-centric trials help to increase patient’s awareness of their own disease, promote continuous learning among health care professionals involved and provide real-world data.

## 2 Methods

### 2.1 Patient journey

**Figure 1:**
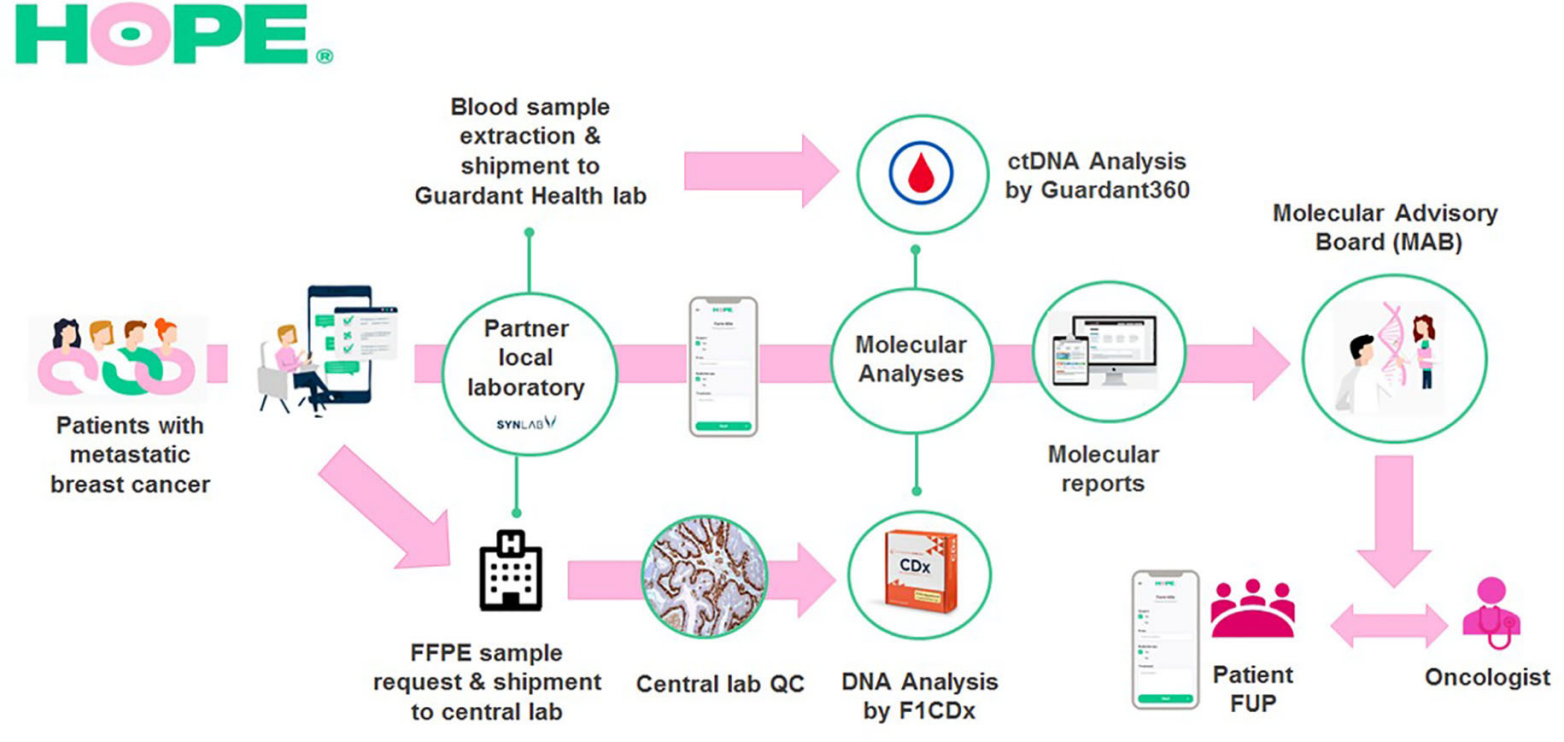
HOPE BC Patient Journey. Patients with metastatic breast cancer voluntarily enroll to HOPE using the study’s digital tool. Then, a dedicated SOLTI team validates inclusion/exclusion criteria and assists them during digital signature of the study informed consent form. Upon inclusion, patients provide their own clinical data and request archival FFPE tumor samples at their treating institutions. Samples are handled at partner local laboratories which ship them to the study’s central laboratory where a histologic quality control is performed. Valid samples are sent for DNA sequencing analysis to FoundationOne dependencies to undergo the FoundationOne^®^ CDx test. In parallel, at disease progression, patients attend to their partner local laboratory to undergo a blood extraction that is sent to Guardant Health dependencies for ctDNA analysis with the Guardant360^®^ panel. Patient’s clinical and molecular data are reviewed by a multidisciplinary board (MAB) that provides further interpretation of molecular results and highlights possible specific treatment options. The MAB report is later shared with the patient and his/her treating oncologist. After report delivery patients complete periodic follow-up questionnaires to assess if they receive recommended therapies and their outcomes.

The HOPE study design aims to minimize burden and inconvenience to patients, make the information accessible, ensure that the trial conduct and data generation are regulatory compliant and support potential improvement to the standard of care. HOPE is a prospective study led by patients diagnosed with mBC who are receiving treatment for their advanced disease in real-world clinical practice conditions, with no limits in prior lines of treatment. The study aimed to include 600 pre- or post-menopausal women or men with mBC and living in Spain. Patients lead their inclusion, participation, and follow-up through a DT that guides them in every step of the journey together with the assistance of the SOLTI Team (**Figure 1**. HOPE BC Patient Journey.). To promote patients’ acquisition of a leading role in the study and the subsequent management of their disease, HOPE implemented a patient empowerment program consisting of in-person and virtual informative workshops about precision oncology, held before and throughout the study. Patients’ oncologists can also participate in their patient’s journey if both parties agree.

SOLTI launched a registration webpage (www.soltihope.com) for the study where patients request participation, provide basic correspondence information and consent to be contacted (See **Supplementary Figure 1**). Upon registration, SOLTI explains the study, what is expected from included patients and the role of the DT. The subsequent steps are managed through this DT, email, and by phone call when necessary. Before informed consent is given, patients answer a short clinical questionnaire to assess whether they meet the inclusion criteria. Eligible patients are included after signing the Informed Consent Form, which can be done either electronically with an advanced electronic signature tool or, when necessary, on paper in a partner local office.

After inclusion, patients are requested to answer a detailed clinical questionnaire. To obtain an accurate clinical record, we recommend them to be supported by their oncologists in this step. Subsequently, a SOLTI dedicated team informs patients how to obtain an archival tumor sample from their reference hospital, preferably the most recent sample, which must be sent together with its corresponding pathology report through a partner local laboratory. Samples are then shipped to the central laboratory for histopathologic quality control and subsequent tissue DNA sequencing. In parallel, patients also learn how to be aware of their disease progression through the study empowerment workshops. When signs of progression are detected, patients inform SOLTI and, after validation of the information through a phone call, they are referred to a partner local laboratory to undergo a blood extraction for ctDNA analysis. Disease progression is validated if patient claims that his/her existing metastatic lesions have grown or there are new lesions, and his/her current treatment will be soon modified. Extractions to patients whose new treatment started more than 21 days before communication are not validated. At the beginning of the study in October 2021, performing blood extractions only at disease progression was not required and there was a low rate of somatic ctDNA detection. In February 2022 this requirement was stablished and there was an increase in the rate of somatic ctDNA detection.

Once the tissue and blood genomic results are available, the patient case is evaluated by the study’s MAB. Before evaluation, patients are asked to update their clinical history to report relevant recent events. The MAB is composed by oncologists, pathologists, molecular biologists, laboratory geneticists and oncologists specialized in hereditary cancer who meet periodically by virtual means to review all available cases. Treating oncologists are always invited to participate in the meetings. The MAB delivers an interpretation of the genomic results and, whenever possible, a recommendation for potential targeted therapies or clinical trials considering the alterations detected and the patients’ clinical history. The report is issued in Spanish and patients are encouraged to share it with their treating oncologists if they did not participate in the discussion.

After MAB report delivery, patients enter the final follow-up phase, where they are asked to fill out periodical follow-up questionnaires every year through the DT for two more years or until patient’s decease.

### 2.2 Sample analysis

Included patients are prompted to deliver their most recent FFPE tumor sample, preferably from metastatic origin, and its corresponding pathology report in one of our partner local laboratories. The HOPE Study provides patients with an informative letter addressed to treating institutions explaining the study and the purpose of the analysis to facilitate archival FFPE tumor sample obtention. Samples obtained are subjected to histopathologic quality control. Acceptable samples include core needle biopsies of deep metastatic lesions; excisional, incisional, or punch biopsies for cutaneous, subcutaneous, or mucosal lesions; or biopsies from bone metastases. If metastatic tissue is not available, breast or metastatic lymph node from the initial surgery can also be analyzed. Fine needle aspiration, brushing, cell pellet from pleural effusion and lavage samples are not accepted. Samples are stained with hematoxylin-eosin and centrally evaluated by a pathologist (not all samples evaluated by the same pathologist). Only samples with a minimal surface of 5mm^2^ and a minimal tumoral percentage of 20% are accepted. Valid samples are shipped to Foundation Medicine^®^, Inc. to undergo the FoundationOne^®^ CDx DNA sequencing panel (F1CDx). This NGS panel includes multiple genes known to be somatically altered in human solid tumors that are validated targets for therapy, either approved or in clinical trials, or that are unambiguous drivers of oncogenesis. The assay interrogates 324 genes as well as 36 introns of genes involved in rearrangements (See **Supplementary Figure 2**). Samples with insufficient DNA quantity (< 50ng) or quality are not suitable for analysis. If tissue samples do not meet histopathological requirements or tissue DNA does not pass quality control, patients are asked to send an additional sample that will follow the above-mentioned requirements. After evaluation, all samples are returned.

Upon disease progression, patients undergo a 20 mL blood extraction in a partner local laboratory using a specific kit provided by Guardant Health^®^, Inc. These samples are immediately sent at room temperature, without special conditions, to the company’s dependencies to be analyzed using the Guardant360^®^ circulating tumor DNA (ctDNA) assay (G360). Cell-free DNA (5-30ng) is extracted from plasma, enriched for targeted regions, and sequenced using NGS. The G360 assay interrogates 74 cancer-associated genes related to somatic alterations, covering point mutations, rearrangements, insertions-deletions and copy number alterations (See **Supplementary Figure 3**).

The F1CDx and the G360 tests are clinically validated in Europe and provide a report that reflects both the identified alterations and the molecular eligibility of patients for targeted therapies approved or under clinical trials. These molecular reports are interpreted by the study’s MAB and used as a reference together with patients’ clinical histories to offer a treatment recommendation in the MAB report, whenever possible (See **Supplementary Figure 4**).

### 2.3 Primary objective and endpoint

The primary objective of this study is to assess in a real-world setting the feasibility of integrating a comprehensive genomic profile into the management of metastatic breast cancer patients. The clinical management of the subsequent lines of treatment (including targeted therapies) after having received the molecular results and the MAB report will be described as the primary endpoint.

### 2.4 Data integration

Patients’ clinical information introduced in the digital tool - upon inclusion, before MAB presentation, and during the study follow-up - is regularly downloaded and entered into SOLTI’s electronic case report form (eCRF). The study medical monitor validates clinical data and contacts patients or involved physicians requesting additional information when necessary. Meanwhile, upon F1CDx or G360 molecular report obtention, the information is immediately shared with the treating oncologist (if participating) and integrated with the eCRF including altered genes, the type of alterations (substitutions, insertions-deletions, copy number alterations and gene fusions/rearrangements along with microsatellite instability status and tumor mutational burden) and their variant allele frequency (if applicable). Histopathological data from the archival tumor samples analyzed is obtained from their corresponding pathology reports and introduced in the database.

Conclusions drawn from each clinical case are described in the MAB report, shared with patients and their oncologists, and integrated with the study eCRF.

### 2.5 Educational program for physicians and patients

To promote the use of targeted therapies and patients’ inclusion in clinical trials, it is of utmost importance that physicians are aware of the availability of therapies and clinical trials inside and outside their institutions. Indeed, one of the aims of the HOPE study is to promote physicians’ education on comprehensive molecular profiling in advanced breast cancer and targeted therapies by sharing molecular reports with them that might spur their search for the optimal treatment option. In line with this, the commercial molecular reports issued within the study do include a list of trials opened in Spain that target the molecular alterations observed. However, it is when discussing each clinical case with other professionals that previous clinical experience can be exchanged and awareness of available trials in other institutions across the country is promoted. Of note, the study is creating a space where physicians can learn and ask for advice from other colleagues on the management of their patients.

We believe that the patient-centric approach of our study will also indirectly stimulate patients’ inclusion in clinical trials since participation is not restricted to a certain geographical area and certain reference centers. Thus, participating in HOPE is an opportunity to overcome inequity in treatment access among patients living in different regions. In fact, any oncologist across the country is susceptible to receive a molecular report from his/her patient participating in the HOPE study. Patients’ empowerment workshops about clinical research will also most likely stimulate knowledge among patients and debates between them and their treating physicians which may as well eventually contribute to their participation in clinical trials.

## 3 Results

**Figure 2:**
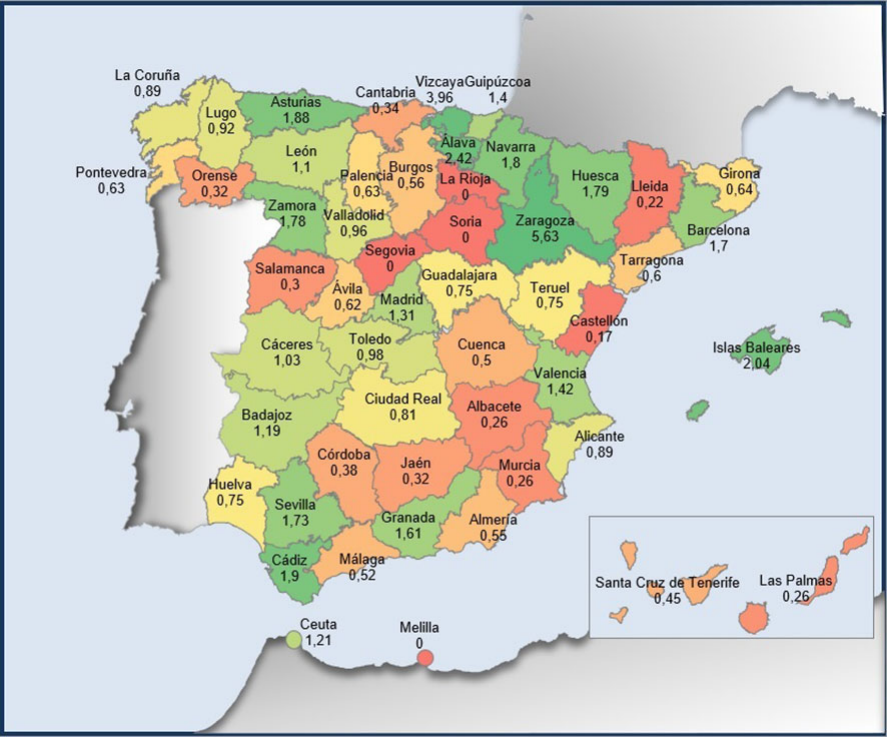
Geographical distribution of patients included in the study per 100.000 inhabitants. Total number of patients included in the study, distributed by regions, and normalized by the population in each region are represented in this map. Patients from almost all Spanish regions have been included. In green, the regions with more representation (≥0.9 patients included in 100.000 inhabitants), in yellow and orange, the regions with intermediate representation (0.9-0,7 and 0,7-0,3, respectively) and in red the less represented regions (<0,3 patients included in 100.000 inhabitants).

The HOPE study was first opened at the end of October 2020. The first estimation was to include 200 patients per year (with an inclusion rate of approximately 17 patients per month) for 3 years. However, at the beginning of March 2021, 344 patients were already participating (inclusion rate of 73 patients per month). At this point, recruitment was stopped to ensure proper management of the available samples and clinical information. In October 2021, recruitment was resumed and completed in February 2022 with a total of 604 patients enrolled (51 patients per month). Patients from almost all Spanish regions have been included. In absolute numbers, the most represented regions are those with the most populated cities (Barcelona, Madrid, Zaragoza, Vizcaya, Valencia, and Sevilla), nevertheless when normalized per the population in each region, the relevance of highly populated areas is reduced showing a more equal distribution (**Figure 2**. Geographical distribution of patients included in the study per 100.000 inhabitants). Most patients included are females (n=601; 99.5%), with only three male patients included (n=3, 0.5%), median age at inclusion was 51 years old (range: 27 to 80), with almost three-quarters of the patients (73.8%) aged between 40 and 60 years old.

**Figure 3:**
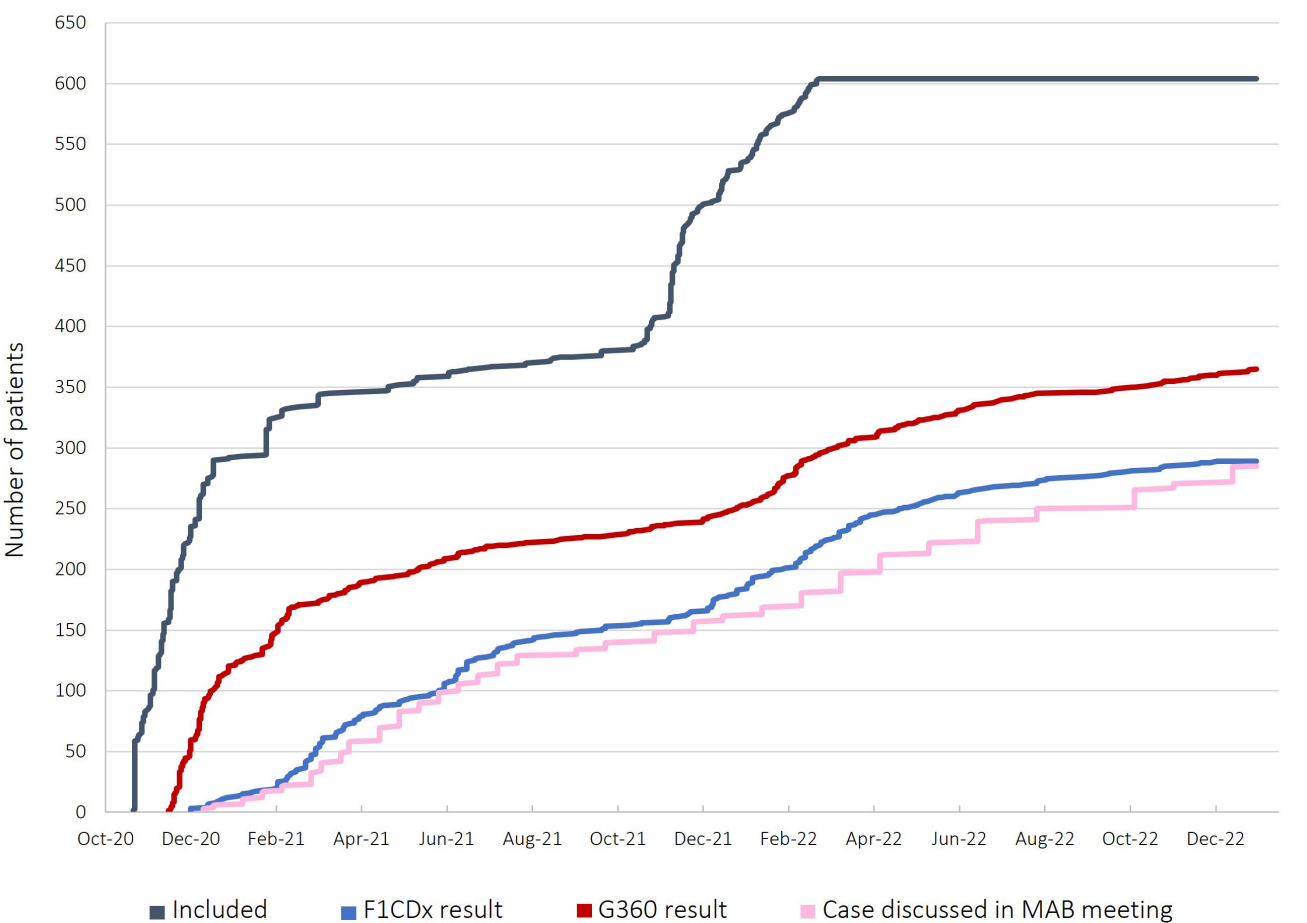
Patient case progression diagram. In grey, the evolution of patient inclusions in the study. Upon study onset, between October and December 2020 several patients were included (more than 300) so the registration was stopped to ensure proper sample analysis. After one year, in October 2021, inclusion was resumed until the maximum number of patients were included (604). In blue, the amount of successful tissue DNA sequencing results obtained with the F1CDx test, which have increased in a constant manner. In dark red, the progression of ctDNA results obtained with the G360 test. From November to February 2021, several G360 results were obtained as disease progression was not required for blood extraction. According to the low rate of somatic ctDNA detection, the requirement for progressive disease was settled and since then G360 results are being obtained more slowly. In pink, the number of patient cases presented in the MAB meetings which is lower than the number of F1CDx results as, to date, only patients with paired analyses are presented in the MAB.

By January 2023, F1CDx data has been obtained from 289 patients that provided solid tumor samples (47.8% of the included) and 365 patients (50.43%) have had a G360 liquid biopsy result. Ultimately, after more than two years since the beginning of the study, 285 patients (47.2%) with paired results or at least with a liquid biopsy result with somatic ctDNA detection, have been presented at the Molecular Advisory Board meetings of the study (**Figure 3**. Patient case progression diagram).

## 4 Conclusion

The HOPE Study is the first prospective and real-world study led by patients diagnosed with mBC in Spain. According to the increasing relevance of NGS technologies and the potential benefit of new targeted therapies in mBC patients’ survival and quality of life, this study aims to evaluate the feasibility and impact of implementing a systematic comprehensive genomic profiling in a population of BC patients. We expect that this collaborative investigational approach, where patients have a central role in almost every step of their journey, will contribute to facilitate patients’ access to tumor and blood genomic sequencing and precision-based medical oncology. Aggregated genomic results will help reflect the mutational status of mBC patients and will show whether this personalized medicine approach is beneficial for the entire population or only for a particular subset. Finally, we strongly believe that the active involvement of patients supported by their oncologists will reinforce patients’ awareness and implication in the management of their disease.

## Data availability statement

The datasets presented in this article are not readily available because of intellectual property restrictions. Requests to access the datasets should be directed to tomas.pascual@gruposolti.org.

## Ethics statement

The studies involving human participants were reviewed and approved by CEIm del Consorci Sanitari del Maresme (Code 62/19). The patients/participants provided their written informed consent to participate in this study.

## Author contributions

All authors participated in the design and/or interpretation of the reported results and participated in the acquisition and/or analysis of data. In addition, all authors participated in drafting and/or revising the manuscript and provided administrative, technical, or supervisory support.
