# Supplementary material for: HOPE (SOLTI-1903) breast cancer study: real-world, patient-centric, clinical practice study to assess the impact of genomic data on next treatment decision-choice in patients with locally advanced or metastatic breast cancer
Source: Front Oncol. 2023 Apr 28;13:1151496. doi: 10.3389/fonc.2023.1151496 (PMC10175800; doi:10.3389/fonc.2023.1151496)
Supplement: Supplementary file 1 [file DataSheet_1.docx]

Supplementary Material

HOPE (SOLTI-1903) Breast Cancer Study: Real-world, patient-centric, clinical practice study to assess the impact of genomic data on next treatment decision-choice in patients with locally advanced or metastatic breast cancer.

Rubén Olivera-Salguero^†^, Elia Seguí^†^, Juan Miguel Cejalvo, Mafalda Oliveira, Pablo Tolosa, Maria Vidal, Marcos Malumbres, Joaquín Gavilá, Cristina Saura, Sonia Pernas, Rafael López, Mireia Margelí, Judith Balmaña, Montserrat Muñoz, Isabel Blancas, Valentina Boni, Eva Ciruelos, Elena Galve, Antonia Perelló, Rodrigo Sánchez-Bayona, Susana de la Cruz, Miguel de la Hoya, Patricia Galván, Esther Sanfeliu, Blanca Gonzalez-Farre, Valeria Sirenko, Aura Blanch-Torras, Jordi Canes, Helena Masanas, Rosa Olmos, Margarita Forns, Aleix Prat, Ana Casas* and Tomás Pascual*.

^†^These authors contributed equally to this work.

*** Correspondence:**Corresponding Authors
[tomas.pascual@gruposolti.org](mailto:tomas.pascual@gruposolti.org); [anacasas@actitudfrentealcancer.org](mailto:anacasas@actitudfrentealcancer.org)

# Supplementary Figures

**
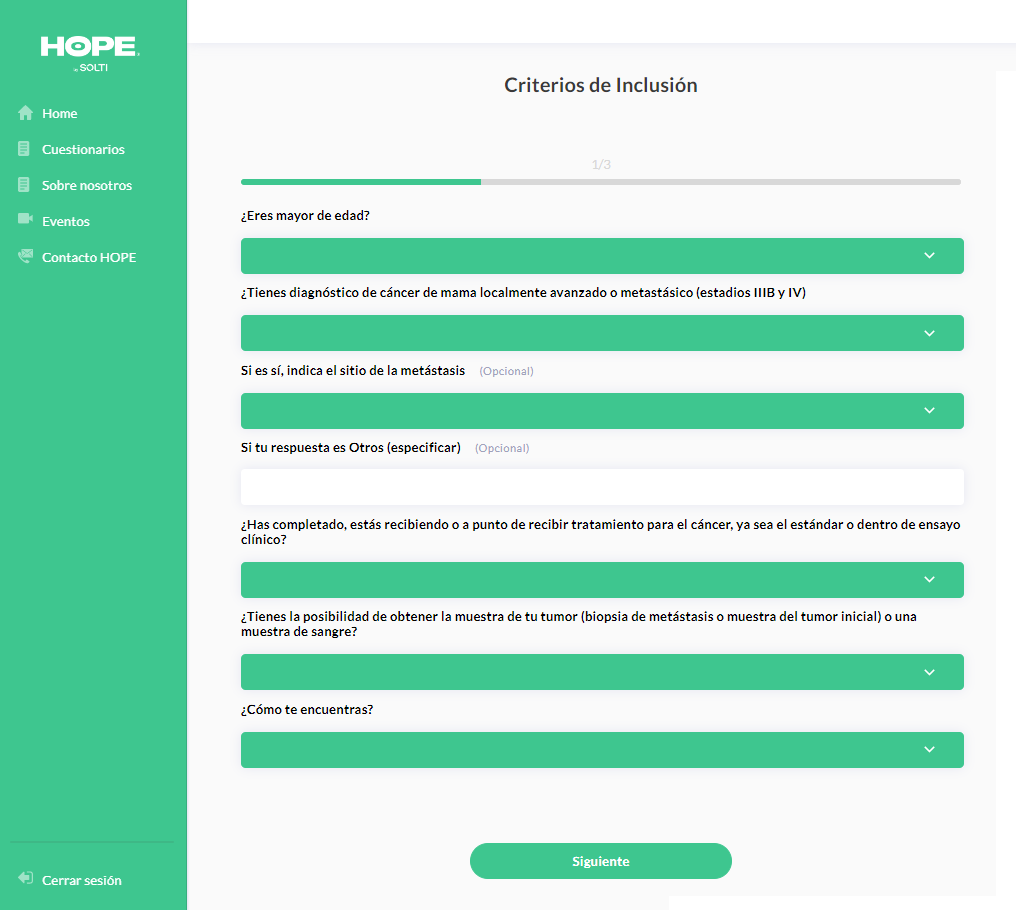
**

**Supplementary Figure 1. Study Digital Tool.** In Spanish, the HOPE (SOLTI-1903) digital tool captures patient information using patient-friendly questionnaires.

**
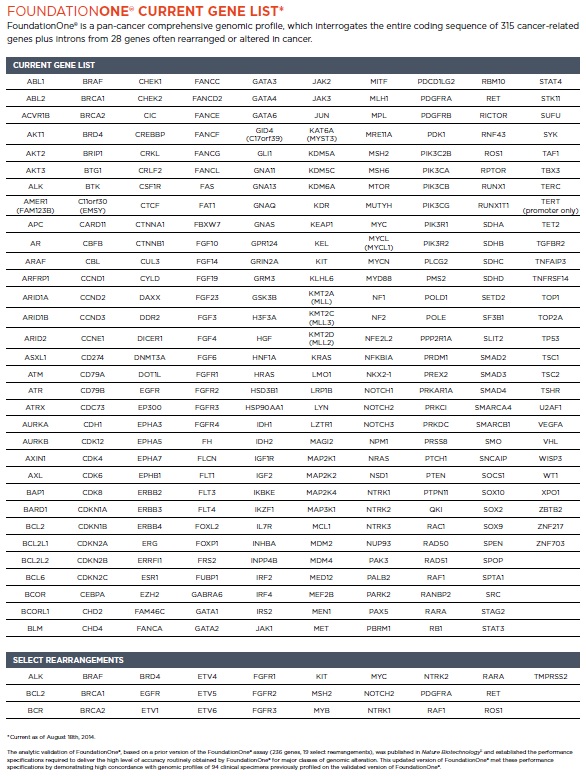
**

**Supplementary Figure 2. F1CDx Gene list.** List of genes interrogated by the FoundationOne® CDx test (19).


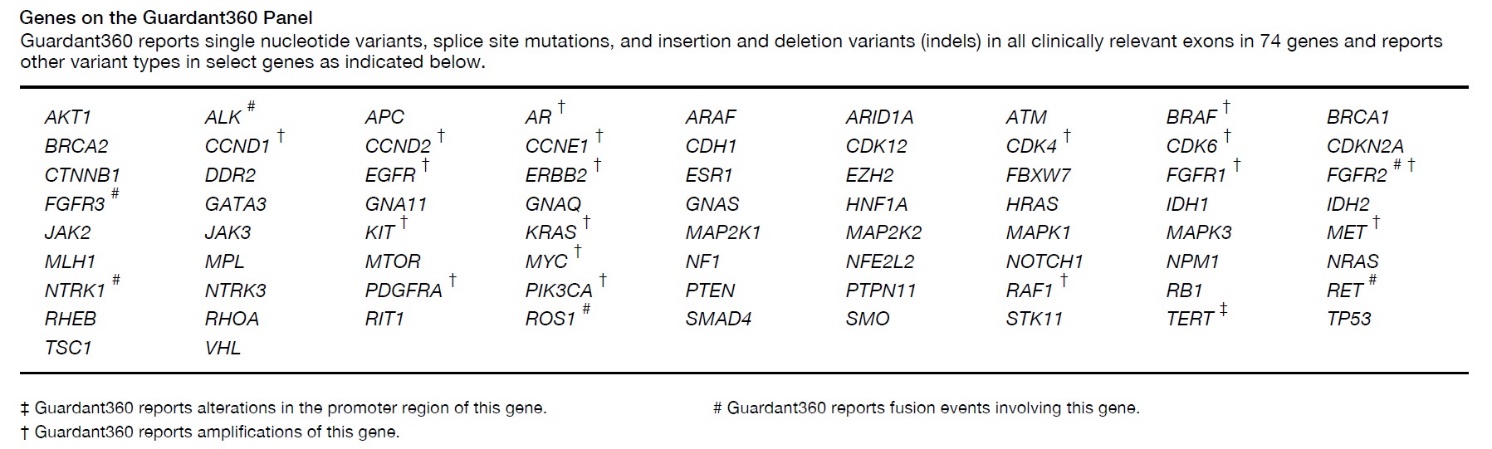


**Supplementary Figure 3. G360 Gene list.** List of genes interrogated by the Guardant360® test (20)

#
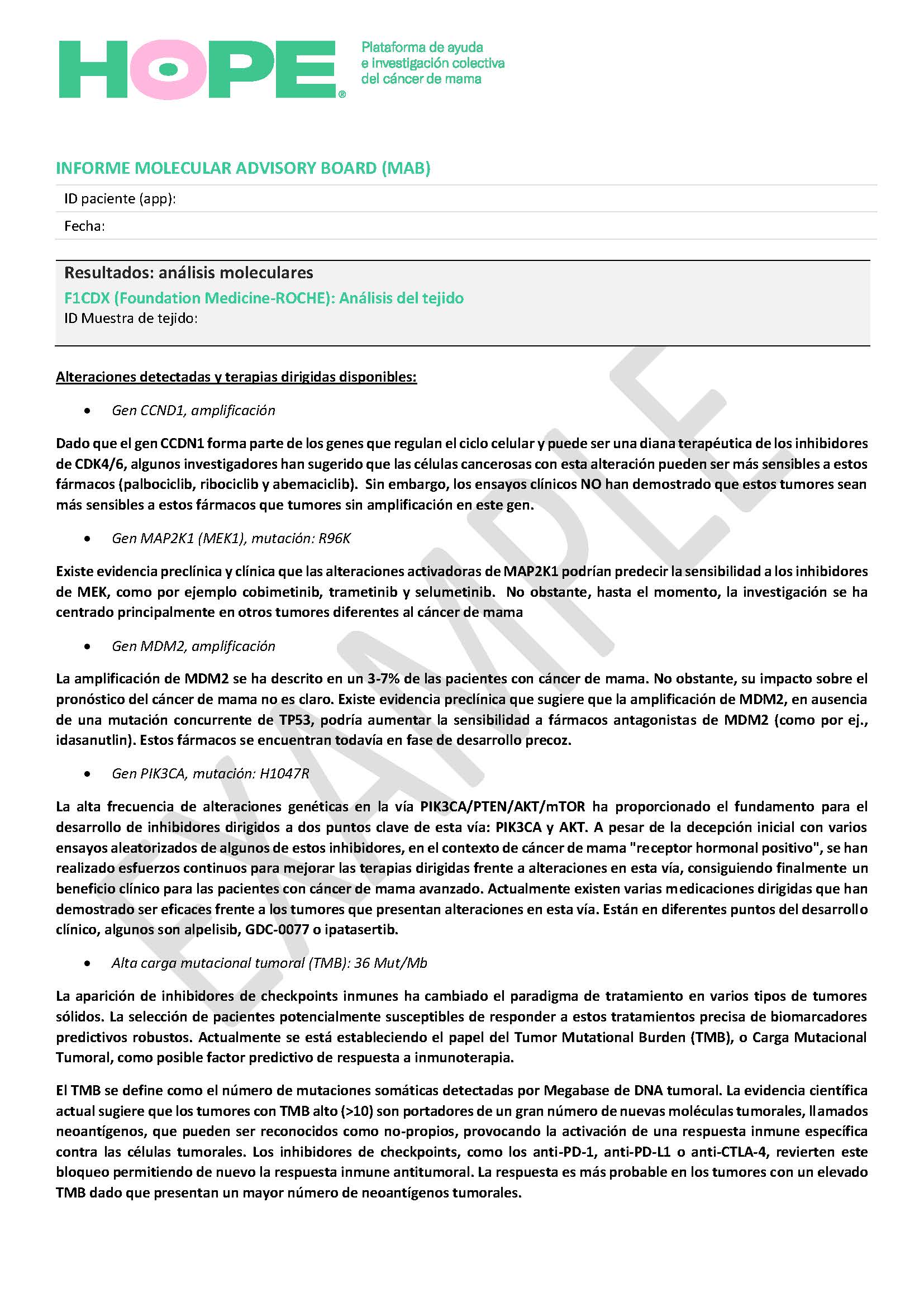


Supplementary Figure 4. Molecular Advisory Board Report page 1 (in Spanish).

#
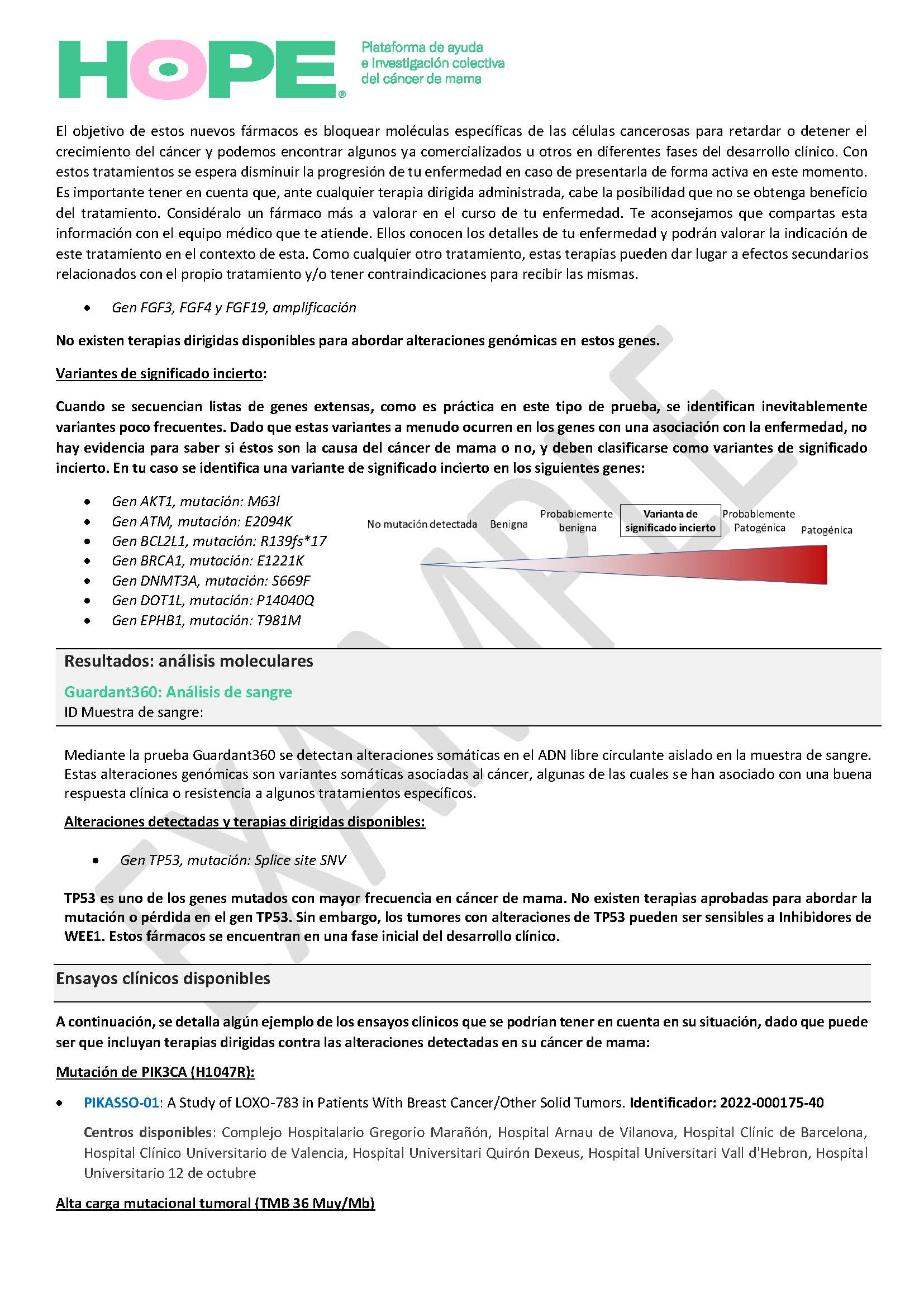


# Supplementary Figure 4. Molecular Advisory Board Report page 2 (in Spanish).

#
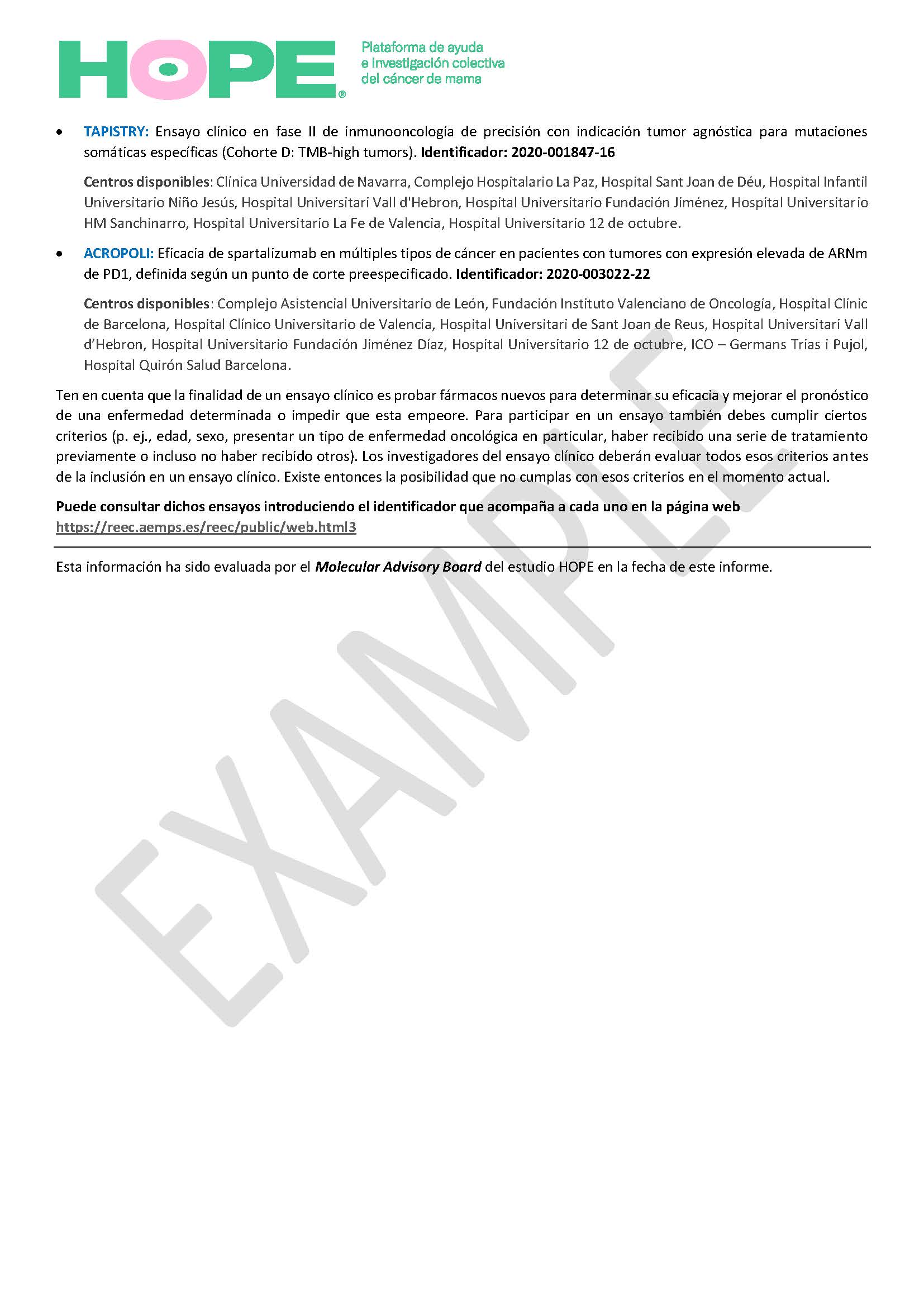
Supplementary Figure 4. Molecular Advisory Board Report page 3 (in Spanish).
